# Inhibitory Effect of *Lactobacillus delbrueckii* subsp. *bulgaricus* KSFY07 on Kappa-Carrageenan-Induced Thrombosis in Mice and the Regulation of Oxidative Damage

**DOI:** 10.1155/2022/4415876

**Published:** 2022-06-15

**Authors:** Pan Wang, Fang Tan, Jianfei Mu, Hongjiang Chen, Xin Zhao, Yanan Xu

**Affiliations:** ^1^Department of Traumatology, Chongqing University Central Hospital & Chongqing Emergency Medical Center, Chongqing 400014, China; ^2^Chongqing Collaborative Innovation Center for Functional Food, Chongqing Engineering Research Center of Functional Food, Chongqing Engineering Laboratory for Research and Development of Functional Food, Chongqing University of Education, Chongqing 400067, China

## Abstract

A mouse thrombosis model was established by kappa-carrageenan to observe the inhibitory effect of *Lactobacillus delbrueckii* subsp. *bulgaricus* KSFY07 (LDSB-KSFY07) on thrombosis and the oxidative stress response. Mouse serum, liver tissue-related indicators, and intestinal microbial composition were measured by examining the expression of microbes in mouse faeces using a biochemical kit, slice observations, and quantitative polymerase chain reaction (qPCR) experiments. The results showed that LDSB-KSFY07 effectively reduced the degree of black tail in thrombotic mice, increased activated partial thromboplastin time (APTT), and decreased thrombin time (TT), fibrinogen (FIB), and prothrombin time (PT) in thrombotic mice. LDSB-KSFY07 was also able to reduce malondialdehyde (MDA) levels and increase superoxide dismutase (SOD), catalase (CAT), and glutathione peroxidase (GSH-Px) levels in the serum and liver tissues of thrombotic mice. Pathological observations showed that LDSB-KSFY07 reduced liver tissue lesions and tail vein thrombosis. Further, experimental results showed that LDSB-KSFY07 was able to upregulate the mRNA expression of copper/zinc-SOD (Cu/Zn-SOD), manganese-SOD, and GSH-Px in the liver tissue of thrombotic mice. Moreover, LDSB-KSFY07 was also able to downregulate the mRNA expression of NF-*κ*B p65, intercellular cell adhesion molecule-1 (ICAM-1), vascular cell adhesion molecule-1 (VCAM-1), and E-selectin in tail vein vascular tissue. Meanwhile, LDSB-KSFY07 could raise plasminogen activator inhibitor-1 (PAI-1) mRNA expression and reduce tissue plasminogen activator (t-PA) expression in heart and tail vein vascular tissues of thrombotic mice. A mouse faeces examination revealed that LDSB-KSFY07 could also upregulate *Bacteroides*, *Lactobacterium*, and *Bifidobacterium* microbial expression and downregulate *Firmicutes* expression in the gut. These results indicate that LDSB-KSFY07 was able to inhibit mouse thrombosis and reduce liver oxidative stress damage in thrombus mice and show that high concentrations of LDSB-KSFY07 provided a better response similar to that of the drug heparin.

## 1. Introduction

Thrombosis is a condition that does great harm to the human body and can affect blood circulation and cardiovascular function [1]. Chronic thrombosis can also cause cerebral tissue ischaemia, hypoxia, softening, and necrosis of cerebral thrombosis [2]. Most cardiovascular and cerebrovascular diseases do not present obvious signs before onset, and if they are not treated in a timely treatment, then death is likely to occur, with cerebrovascular diseases presenting a very high mortality rate [3]. Thrombosis is a great threat to human health that has attracted increasing attention. However, many drugs for the treatment of thrombosis have obvious side effects. The prevention and intervention effects on thrombosis of side effect-free biological strategies have become a hot topic of research [4]. Intraperitoneal injection of kappa-carrageenan has been shown to cause oxidative stress and inflammation in experimental animals, and the release of a large number of inflammatory factors and free radicals into the blood can damage vascular endothelial cells and cause thrombosis. Moreover, because blood flow to the mouse tail occurs through a single femoral artery, an embolism in this artery generally does not cause gradual ischaemia, and necrosis of mouse tail tissue occurs after collateral circulation is engaged [5]. In this study, an animal thrombosis model was established by kappa-carrageenan to stimulate oxidative stress inflammation leading to tail vein thrombosis in mice.

Inflammation induces thrombosis, which further exacerbates inflammatory development, thus representing a vicious cycle called a “thrombotic inflammatory response.” Both the occurrence and development of thrombosis and inflammation are accompanied by reactive oxygen species (ROS) production and oxidative stress damage, which leads to the failure of the “oxidative stress-inflammation-thrombosis” cycle [6]. Thrombotic inflammation can extend to the body and damage distal organs, such as the heart, lungs, and brain, thus leading to multiorgan dysfunction and death. After the release of inflammatory factors, blood becomes hypercoagulant, which induces thrombosis [7]. Inflammation is closely linked to oxidative stress. ROS are important mediators of the inflammatory response, which promotes ROS production and causes oxidative stress; in particular, superoxide anion radical, hydroxyl radical, and hydrogen peroxide radical play the key roles [8]. Injury of the vascular endothelium by ROS-induced oxidative stress is an important factor in inducing and exacerbating thrombosis. When stimulated, the vascular endothelium produces a large amount of ROS, and high levels of ROS result in impaired vessels and increased adhesion molecules and stimulate and aggravate inflammation, thus leading to thrombosis. Oxidative stress induces thrombosis, while thrombosis further exacerbates oxidative stress [9].

The Xinjiang Uygur Autonomous Region is located in the southwest frontier region of China and has a vast terrain, and animal husbandry is the local pillar industry. There are many ethnic minorities in Xinjiang, and most of them drink dairy products. Homemade fermented yogurt is one of the most common dairy products and represents an indispensable food in the daily life of ethnic minorities [10]. Compared with commercially produced yogurt, the developed animal husbandry and years of natural fermentation associated with the production process of fermented acid milk in Xinjiang is more ecological and natural, the types of probiotics are more abundant, and the acidified milk has more prominent nutritional value and health functions [11, 12]. After thousands of years of natural domestication, the microorganisms in these fermented dairy products have adapted to the living environment of Xinjiang and have different fermentation characteristics and probiotic functions [12]. Lactic acid bacteria isolated from Xinjiang traditional fermented acid milk have good biological activity and good antioxidant effects [13], and they can also prevent obesity caused by a high-fat diet [14] and have a certain preventive effect on colitis and colon cancer [15]. Therefore, lactic acid bacteria can be used in probiotic preparations. This study investigated lactic acid bacteria isolated from Xinjiang naturally fermented yogurt (LDSB-KSFY07); it is a safe and nontoxic microorganism [12]. The intervention effect of lactic acid bacteria on thrombosis by regulating the level of oxidative stress and inflammation can be observed using the mouse tail thrombosis model to explore new strategies that incorporate probiotics to prevent thrombosis, assist in eliminating thrombosis, and reduce the risk of various diseases caused by thrombosis.

## 2. Materials and Methods

### 2.1. Experiment Strain

LDSB-KSFY07 was isolated from naturally fermented yogurt in Kashgar, Xinjiang, China. The strain was identified as *Lactobacillus delbrueckii* subsp. *bulgaricus* (GACATCTGCTGTCTTAGGCGGCTGACTCCTATAAAGGTTATCCCACCGACTTTGGGCATTGCAGACTTCCATGGTGTGACGGGCGGTGTGTACAAGGCCCGGGAACGTATTCACCGCGGCGTGCTGATCCGCGATTACTAGCGATTCCAGCTTCGTGCAGGCGAGTTGCAGCCTGCAGTCCGAACTGAGAACAGCTTTAAGAGATCCGCTTACCCTCGCGGGTTCGCTTCTCGTTGTACTGCCCATTGTAGCACGTGTGTAGCCCAGGTCATAAGGGGCATGATGACTTGACGTCATCCCCACCTTCCTCCGGTTTGTCACCGGCAGTCTCTTTAGAGTGCCCAACTTAATGATGGCAACTAAAGACAAGGGTTGCGCTCGTTGCGGGACTTAACCCAACATCTCACGACACGAGCTGACGACAGCCATGCACCACCTGTCTCTGCGTCCCCGAAGGGAACCACCTATCTCTAGGTGTAGCACAGGATGTCAAGACCTGGTAAGGTTCTTCGCGTTGCTTCGAATTAAACCACATGCTCCACCGCTTGTGCGGGCCCCCGTCAATTCCTTTGAGTTTCAACCTTGCGGTCGTACTCCCCAGGCGGAGCGCTTAATGCGTTTGCTGCGGCACTGAGAACCGGAAAGTCCCCAACACCTAGCGCTCATCGTTTACGGCATGGACTACCAGGGTATCTAATCCTGTTCGCTACCCATGCTTTCGAGCCTCAGCGTCAGTTGCAGACCAGAGAGCCGCCTTCGCCACTGGTGTTCTTCCATATATCTACGCATTCCACCGCTACACATGGAATTCCACTCTCCTCTTCTGCACTCAAGAATGACAGTTTCCGATGCAGTTCCACGGTTGAGCCG); it was eventually identified and placed in the China General Microbiological Culture Collection Centre (Beijing, China) under retention number CGMCC 15714.

### 2.2. Mouse Thrombotic Model

Fifty 6-week-old specific pathogen free- (SPF-) grade Institute of Cancer Research (ICR) male mice weighing 23 ± 2 g were purchased from the Laboratory Animal Centre, Chongqing Medical University (Chongqing, China). The conditions met the rearing requirements and other experimental operations met the ethical requirements of experimental animals. Fifty ICR mice were randomly divided into five groups, including normal, model, heparin (drug positive control), LDSB-KSFY07 low concentration (LDSB-KSFY07-L), and LDSB-KSFY07 high concentration (LDSB-KSFY07-H) groups; there were 10 mice in each group. Normal mice were injected intraperitoneally with saline solution (0.1 mL solution was injected every 10 g of mouse body weight) daily, and the remaining mice were injected with 0.2% kappa-carrageenan (CAS No: 11114-20-8, C_24_H_36_O_25_S_2_, 0.1 mL solution was injected every 10 g of mouse body weight) solution for 10 days [16, 17]. Meanwhile, mice in the heparin group were treated with heparin daily at subcutaneous injection at 5000 U/kg body weight, and mice in the low and high LDSB-KSFY07 groups were gavaged daily with LDSB-KSFY07 at 10^8^ CFU/kg body weight and 10^9^ CFU/kg body weight, respectively, for 10 days. The length of black tail (thrombus) in each group was recorded 10 days later. And the mice were sacrificed by cervical dislocation.

### 2.3. Determination of Four Parameters of Hemagglutination

The collected mouse blood was placed in a centrifuge tube, and 100 *μ*L blood preheated at 37°C for 3 min and 200 *μ*L PT reagent preheated at 37°C for 10 min were added to the semiautomatic coagulation instrument (PUN-2048A, Beijing Pulang New Technology Co., Ltd., Beijing, China) for PT determination. For APTT measurement, 37°C and 3 min prewarmed 100 *μ*L of blood and APTT reagent were added to the analyzer, and then, 100 *μ*L of saturated CaCl_2_ solution preheated at 37°C for 10 min was added for the measurement. During TT measurement, 100 *μ*L blood preheated at 37°C for 3 min and 100 *μ*L TT solution preheated at 37°C for 10 min were added to the analyzer for measurement. For FIB measurement, 20 *μ*L of plasma preheated at 37°C for 3 min was added to the analyzer, then 180 *μ*L of IBS was added, and then, 100 *μ*L of FIB solution preheated at 37°C for 10 min was added for the determination.

### 2.4. Determination of the Oxidation Factor Index

Blood serum was obtained by centrifugation of the extracted whole blood sample at 4000 rpm and 4°C for 10 min. In addition, 0.1 g of liver tissue was precisely weighed, with 0.9 mL of saline added for homogenization. The homogenate was collected by centrifugation at 4000 rpm, and the supernatant was collected at 10 min. The SOD, CAT, GSH-Px enzyme activities, and MDA level of the serum and liver tissue were determined using assay kits (Nanjing Jiancheng Bioengineering Institute, Nanjing, Jiangsu, China) [18].

### 2.5. Histopathological Observations

Liver tissue, tail tissue, and cardiac tissue collected after dissection were fixed in 10% formalin, and after 48 h of dehydration, the tissue samples were subjected to paraffin embedding, sectioning, haematoxylin-eosin (H&E) staining, and tissue pathology observations by optical microscopy (BX43, Olympus, Tokyo, Japan) [19].

### 2.6. Determination of Tissue mRNA Expression

Mouse tail, liver, and heart tissue specimens with a precise weight of 0.2 g were homogenized with 9 mL of saline, and then, 0.5 mL of RNAzol (Invitrogen, New York, USA) was added to extract RNA. The absorbance values of the extracted RNA were measured at 260 nm and 280 nm using ultradifferential photometry, the RNA purity and concentration were calculated, and the RNA concentration was adjusted to 1 *μ*g/*μ*L (Nano-300, Hangzhou Allsheng Instruments Co., Ltd., Zhejiang, China). Then, after generating cDNA by inversion, a reaction system containing 1 *μ*L cDNA was prepared. The remaining reagents included 10 *μ*L SYBR Green PCR Master Mix, 7 *μ*L of sterile distilled water, and a 1 *μ*L primer solution (forward and reverse sequences) (Table 1, Thermo Fisher Scientific, Waltham, MA, USA). Reactions were performed using a quantitative PCR instrument (StepOne Plus, Thermo Fisher Scientific) with the following reaction conditions: 95°C for 60 s, 95°C for 15 s for 40 cycles, 55°C for 30 s, 72°C for 35 s, 95°C for 30 s, and 55°C for 35 s. *β*-Actin was selected as the internal reference, and its associated genes were analyzed by the 2^−*ΔΔ*Ct^ method [20].

### 2.7. Determination of Microbial mRNA Expression

One gram of mouse faeces was accurately weighed and homogenized with 9 mL saline. Microbial mRNA expression in the mouse faeces was then determined by the tissue mRNA assay as described above to detect the microbial composition in the faeces.

### 2.8. Statistical Analysis

All experiments were performed in parallel three times, and the results were used to determine the mean. The standard deviation was then calculated, and the experimental results were expressed as the mean ± standard deviation. One-way ANOVA was also calculated for significant differences between data groups by Duncan's multiple range test (*P* < 0.05).

## 3. Results

### 3.1. Level of Black Tail in Mice

**Figure 1:**
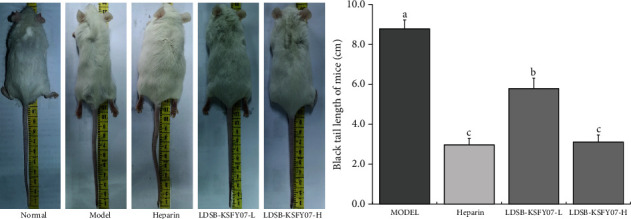
Tail thrombus of mice in each group. The results have been expressed as mean ± deviation. (A–D) Mean values with different bars in the different column are significantly different (*P* < 0.05) according to Duncan's multiple range test.

After intraperitoneal injection with kappa-carrageenan, the tip of the black tail showed thrombosis (Figure 1). The tail of the mouse was dissected and gradually cut from the position close to the body until a clotting spot was observed and identified as the location of the black tail. The model group mice had the longest black tail length (8.78 ± 0.44 cm), which was significantly higher than that in the remaining groups (*P* < 0.05). The black tail of mice in LDSB-KSFY07-H group was shorter (3.12 ± 0.35 cm), which was similar to that of the heparin group (2.97 ± 0.32 cm) and significantly shorter than that of the LDSB-KSFY07-L group (3.12 ± 0.35 cm, *P* < 0.05).

### 3.2. Four Hemagglutination Parameters in Mice

The normal group of mice had a significantly higher APTT but significantly lower TT, FIB, and PT than the other groups (*P* < 0.05, Table 2). The model group presented the opposite situation and showed significantly lower APTT and significantly higher TT, FIB, and PT than the other groups (*P* < 0.05). The LDSB-KSFY07-H group presented significantly higher APTT and significantly lower TT, FIB, and PT than the LDSB-KSFY07-L group (*P* < 0.05). The APTT, TT, FIB, and PT levels in the LDSB-KSFY07-H group were close to those in the heparin group, with no significant difference (*P* > 0.05).

### 3.3. Oxidation Index of Mice

The serum and liver tissue analyses showed that the SOD, CAT, and GSH-Px levels of model group mice were highest and the MDA level was lowest (Table 3). The SOD, CAT, and GSH-Px enzyme activities in the heparin and LDSB-KSFY07-H groups were slightly lower than those in the normal group and higher than those in the LDSB-KSFY07-L group, and the SOD, CAT, and GSH-Px enzyme activities in the model group were the lowest (*P* < 0.05). The model group had the highest level of MDA, the LDSB-KSFY07-L group presented a lower level, and the heparin and LDSB-KSFY07-H groups presented significantly lower levels (*P* < 0.05).

### 3.4. Pathological Observations of Liver, Tail Vein, and Heart Tissues in Mice

**Figure 2:**
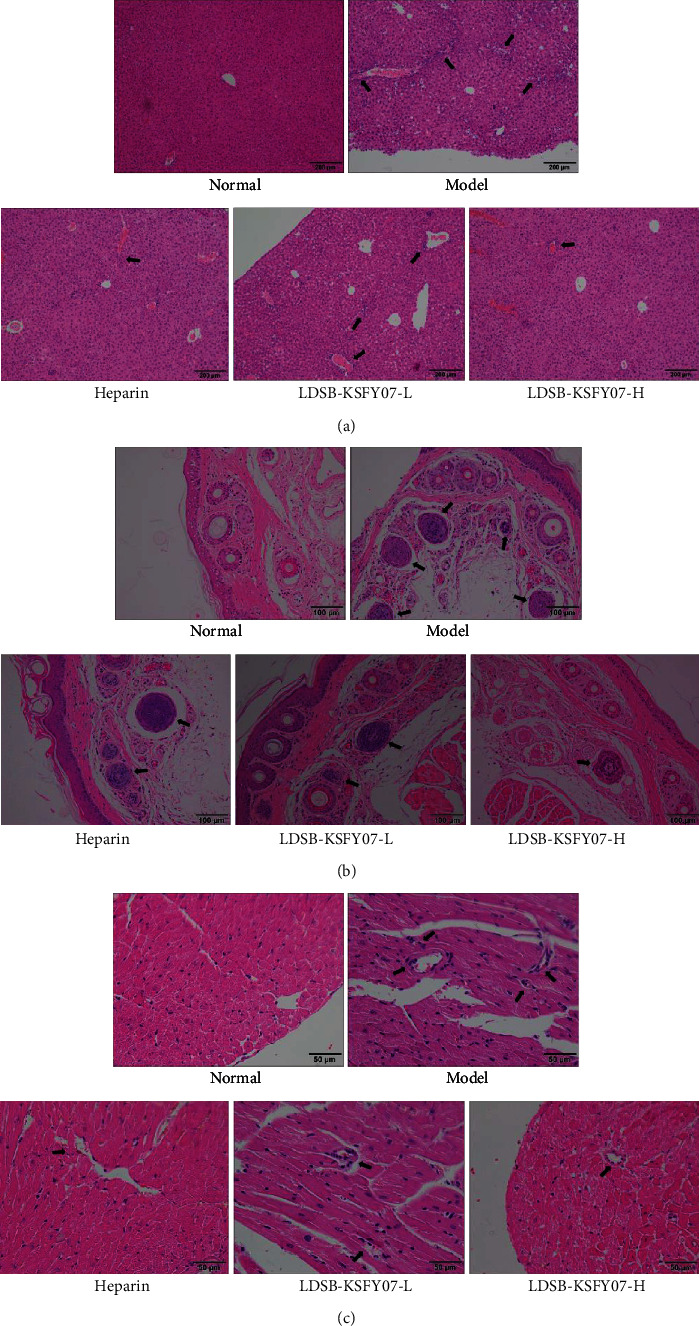
Pathological observation of liver (a), tail (b), and cardiac (c) tissues in mice with kappa-carrageenan-induced thrombosis. The arrow indicates the site of tissue lesion.

The hepatocytes in the liver of the normal group mice were complete, showed a clear structure, and were evenly distributed in normal sections (Figure 2(a)). Sections of the model group showed severe hepatocyte necrosis and inflammatory cell infiltration, with an irregular arrangement of hepatocytes around the central vein. After the remaining groups of mice were treated with heparin and LDSB-KSFY07, the liver tissue damage was relieved, with heparin and LDSB-KSFY07-H showing a better response and producing a liver tissue morphology close to the normal state.

The vessel morphology of the tail vein was rounded and the vessel wall was smooth in the normal group (Figure 2(b)). In the model group, leukocyte infiltration and inflammatory exudation were observed in the blood tube wall of the tail vein, and bleeding lesions, platelet aggregation, and thrombosis were observed in the blood vessels. Both LDSB-KSFY07 and heparin were able to reduce the lesions of the tail vein vessels in mice, with LDSB-KSFY07-H and heparin showing similar effects and performing better than LDSB-KSFY07-L.

In the normal group, the transverse lines of the inner muscle cells in the heart tissue were clear, the nucleus was located in the middle, and there were dotted radial myofibrils or neatly arranged myocardial fibers around the nucleus, and there was no myocardial edema, blood stasis, degeneration, inflammatory cells, neutrophils, and other infiltration (Figure 2(c)). In the model group, the boundary of cardiomyocytes was unclear, and the volume increased myocardial fibrosis, partial calcification, inflammation, and central granulocyte infiltration. LDSB-KSFY07 and heparin could improve the heart disease caused by thrombosis. Most of the myocardial fibers in the LDSB-KSFY07-H group, LDSB-KSFY07-L group, and heparin group were intact, and most of the myocardial cells had clear boundaries without inflammatory infiltration. However, some cardiomyocytes were still calcified in the LDSB-KSFY07-L group.

### 3.5. mRNA Expression in the Mouse Liver Tissues

**Figure 3:**
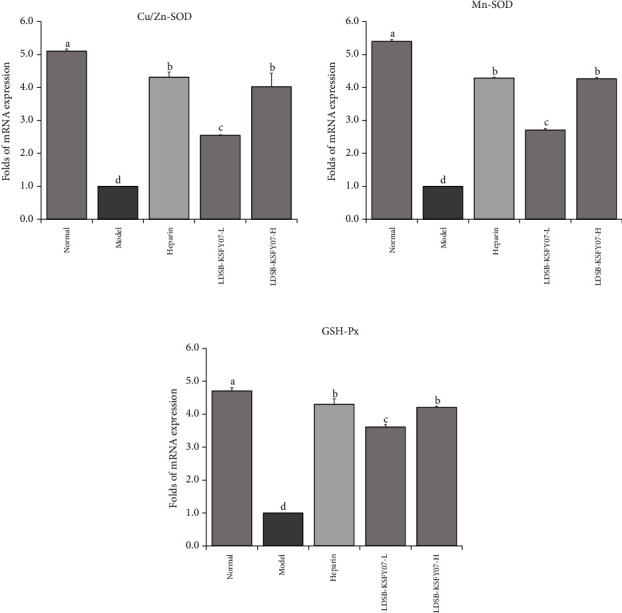
Cu/Zn-SOD, Mn-SOD, GSH-Px, and MDA mRNA expressions in liver tissue of mice with kappa-carrageenan-induced thrombosis. The results have been expressed as mean ± deviation. (A–D) Mean values with different bars in the different column are significantly different (*P* < 0.05) according to Duncan's multiple range test.

The Cu/Zn-SOD, Mn-SOD, and GSH-Px mRNA expression in the liver tissues of the normal group was significantly stronger than that in the other groups (Figure 3, *P* < 0.05). Mice in the model group expressed the weakest Cu/Zn-SOD, Mn-SOD, and GSH-Px. Both LDSB-KSFY07 and heparin were able to upregulate Cu/Zn-SOD, Mn-SOD, and GSH-Px expression in the liver tissue of thrombotic mice. Moreover, LDSB-KSFY07-H and heparin presented similar effects, with both significantly better than LDSB-KSFY07-L (*P* < 0.05).

### 3.6. mRNA Expression in Mouse Tail Vein Tissues

**Figure 4:**
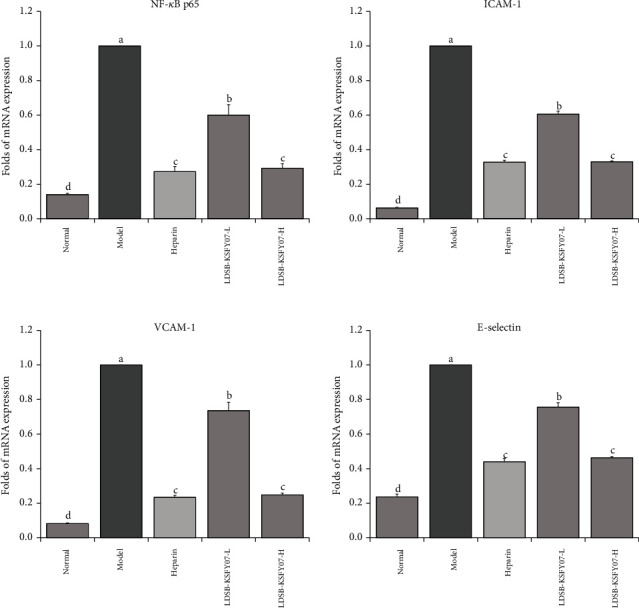
NF-*κ*B p65, ICAM-1, VCAM-1, and E-selectin mRNA expressions in tail vein tissue of mice with kappa-carrageenan-induced thrombosis. The results have been expressed as mean ± deviation. (A–D) Mean values with different bars in the different column are significantly different (*P* < 0.05) according to Duncan's multiple range test.

NF-*κ*B p65, ICAM-1, VCAM-1, and E-selectin expressions were strongest in the tail vein vessels of the model group (Figure 4). LDSB-KSFY07-L, LDSB-KSFY07-H, and heparin were able to significantly downregulate NF-*κ*B p65, ICAM-1, VCAM-1, and E-selectin expression in the tail vein vessels of thrombotic mice (*P* < 0.05), with the resulting expression close to that of the normal group of mice. Moreover, the differences in expression in the LDSB-KSFY07-H and heparin groups were not significantly different, although both groups showed slightly higher expression than the normal group.

### 3.7. Coagulation mRNA Expression in the Mouse Heart and Mouse Tail Vein Tissues

**Figure 5:**
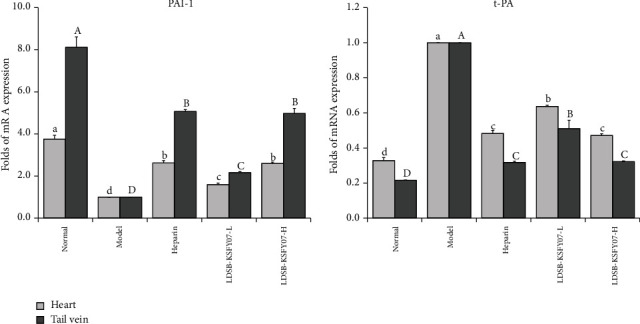
TAI-1 and t-PA mRNA expression in heart and tail vein tissues of mice with kappa-carrageenan-induced thrombosis. The results have been expressed as mean ± deviation. (a–d and A–D) Mean values with different bars in the different column are significantly different (*P* < 0.05) according to Duncan's multiple range test.

After thrombus induction (model group), the mRNA expression of PAI-1 in mice heart and mouse tail vein tissues decreased (Figure 5), while the expression of t-PA increased. Compared with the model group mice, LDSB-KSFY07-L, LDSB-KSFY07-H, and heparin could raise the PAI-1 expression and reduce the t-PA expression. LDSB-KSFY07-H and heparin could make PAI-1 and t-PA expression close to the normal group.

### 3.8. Microbial mRNA Expression in Mouse Faeces

**Figure 6:**
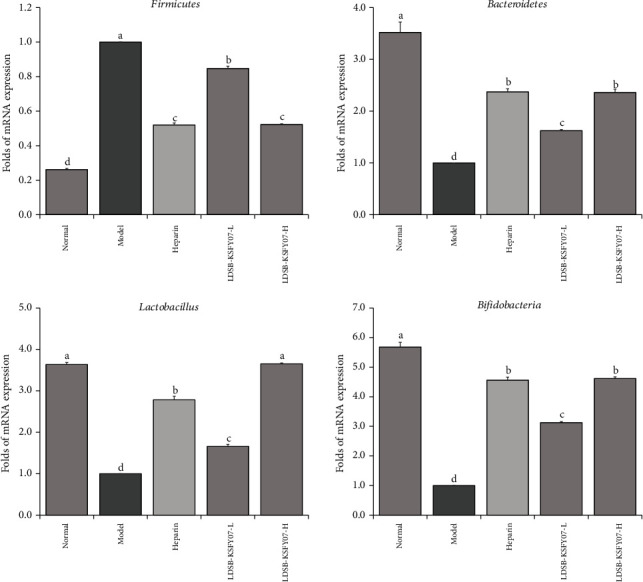
The mRNA expression of *Firmicutes*, *Bacteroidetes*, *Lactobacillus*, and *Bifidobacteria* in faeces of mice with kappa-carrageenan-induced thrombosis. The results have been expressed as mean ± deviation. (A–D) Mean values with different bars in the different column are significantly different (*P* < 0.05) according to Duncan's multiple range test.

The mRNA expression of *Firmicutes* microorganisms in the stool of normal mice was significantly lower than that in the other groups, whereas *Bacteroides* and *Bifidobacterium* were significantly higher than those in the other groups (Figure 6, *P* < 0.05). After induction of thrombosis, the model group showed the highest *Firmicutes* mRNA expression but lowest in *Bacteroides*, *Lactobacterium*, and *Bifidobacterium* expression. LDSB-KSFY07 and heparin were able to reduce the expression of *Firmicutes* in the stool of thrombus mice and enhance the expression of *Bacteroides* and *Bifidobacterium*. In addition, there was no significant difference in *Lactobacterium* expression between the normal and LDSB-KSFY07 groups, although the expression was significantly higher in these two groups than the other groups (*P* < 0.05).

## 4. Discussion

Kappa-carrageenan can lead to the formation of thrombosis associated with endovascular oxidative stress damage in the mouse tail, which results in the filling of venules, arterioles, and capillaries in the mouse tail with mixed thrombi and subsequent ischaemia and necrosis of the tail tissue based on intuitive observations of black tail in mice [21]. Therefore, mouse black tail length is an important experimental measure to intuitively determine the degree of thrombosis. This study again demonstrates that kappa-carrageenan can lead to obvious mouse black tail formation and shows that both heparin and LDSB-KSFY07 can reduce the black tail caused by thrombosis. Moreover, a higher concentration of LDSB-KSFY07 produces a better response that is close to the common antithrombotic drug heparin.

The PT, APTT, TT, and FIB index test pairs are clinical indicators for the diagnosis of haematological diseases with abnormal coagulation systems [22]. Consumption of numerous coagulation factors during thrombosis causes prolonged PT, while the loss of coagulation factors causes shortened APTT [23]. Under the action of thrombin, FIB is constantly converted into fibrin, the main component of thrombosis, which maintains a high coagulation state of blood. In the presence of thrombus, especially when thrombus affects liver function, FIB will increase abnormally and is also an important factor to observe the influence of thrombus [24]. When the blood fibrin content is too high, the body enhances fibrinolysis, which increases fibrin degradation products, resulting in prolonged TT [25]. Prolonged APTT reflects the lack of clotting factors associated with endogenous clotting pathways, and prolongation of PT reflects the lack of coagulation factors related to exogenous clotting pathways [22]. In the absence of coagulation factors, TT will also increase, and when ATPP and PT have significant changes, TT will also change. With the intervention of external therapeutic agents, TT will be immediately shortened, which can clearly show the effect [25]; these changes are important clinical indicators of thrombus and coagulation status monitoring. The experimental results of this study are similar to those obtained by current clinical tests; heparin and LDSB-KSFY07 were able to control four indicators of blood coagulation, implying that heparin and LDSB-KSFY07 can intervene in thrombosis; moreover, the effect of LDSB-KSFY07 was enhanced as its concentration increased.

Free radical accumulation is an important factor that induces and exacerbates thrombosis. ROS can not only directly activate platelets but also significantly improve the sensitivity to platelet aggregates, such as thrombin, collagen, and arachidonic acid. Moreover, ROS released by leukocytes can help to induce platelet and leukocyte aggregation and promote thrombosis [26]. ROS can also activate NF-*κ*B signalling to mediate endothelial apoptosis and promote thrombus molecular secretion, leading to the formation of venous thrombosis [27]. Studies have shown that free radical scavengers can completely prevent iron ion-induced thrombosis, fully reflecting the important role of oxidative stress in thrombosis [28]. SOD can catalyse superoxygen anion free radical disambiguation to generate oxygen and hydrogen peroxide and plays a crucial role in the balance of body oxidation and antioxidant oxidation, and Cu/Zn-SOD and Mn-SOD are the two important classes present in mammals [29]. CAT is an enzymatic scavenger that drives the breakdown of H_2_O_2_ into molecular oxygen and water, and its enzymatic activity provides an antioxidant defence for the body [30]. Both SOD and CAT act as antioxidant enzymes that can prevent ROS from causing damage to the body, and they are also effective active substances that can reduce the oxidative stress that causes thrombosis [31]. GSH-Px is an important peroxide catalase that can protect the structure and function of the cell membrane and avoid vascular interference and damage by peroxides [32]. Within organisms, free radicals act on lipid peroxidation. The oxidation end product of MDA will cause crosslinking polymerization of vital macromolecules, such as proteins and nucleic acids, and this process is cytotoxic. Therefore, the amount of MDA can reflect the degree of lipid peroxidation in the body and indirectly reflect the extent of cellular damage, and its level may also serve as an important hint of thrombosis [33]. In this study, the induction of thrombosis in mice led to increased levels of oxidative stress within the body, decreased the SOD, CAT, and GSH-Px antioxidant enzyme levels and mRNA expression, and increased the MDA levels, which is consistent with the results of previous studies, suggesting that thrombosis is closely associated with oxidative stress; these experimental results are similar to those obtained in other studies above [29–33]. At high concentrations, LDSB-KSFY07 can alter these oxidation-related metrics so that they are close to that of normal mice, with the effect reaching the level of antithrombotic agents.

NF-*κ*B is key to the inflammatory mechanism in deep vein thrombosis, and it disrupts the balance of endothelial cells and inflammatory responses by mediating the interaction between platelets and inflammatory reactions [34]. ICAM-1 mainly mediates the cell-to-cell and cell-to-matrix adhesion responses and plays a significant role in the occurrence and development of inflammation [35]. VCAM-1 can negatively regulate platelet adhesion aggregation and induce inflammatory responses at the clot site [36]. E-selectin can mediate the local adhesion of leukocytes to vascular endothelial cells in a blood flow state, induce inflammatory endothelial damage, increase endothelial cell permeability, and accelerate leukocyte infiltration [37]. The NF-*κ*B signalling pathway is the central link of multiple inflammatory reactions that upregulate the expression of proinflammatory factors under an activated state. In such a state, the NF-*κ*B pathway activates endothelial cells, thus leading to the enhanced expression of adhesion molecules and cytokines, such as ICAM-1, VCAM-1, and E-selectin, which further activate NF-*κ*B, thereby amplifying the inflammatory response, activating platelet aggregation and the coagulation response, and forming a hypercoagulant state [38]. In this study, thrombosis led to oxidative stress damage in the mouse tail vein, which led to an inflammatory response that included an NF-*κ*B-centred pathway in which the associated expression of ICAM-1, VCAM-1, and E-selectin was significantly different from that in the normal state. Both heparin and LDSB-KSFY07 function in regulating NF-*κ*B, ICAM-1, VCAM-1, and E-selectin expression and hence have a good thrombotic effect.

PAI-1 is the main inhibitor of plasma fibrinolytic activity in the body. It regulates and maintains the dynamic balance between fibrinolytic system and coagulation system. Clinical and experimental tests have found that thrombosis will directly reduce the level of PAI-1, resulting in the imbalance between fibrinolytic system and coagulation system [39]. t-PA can also play an important role in the fibrinolytic system. It can inhibit platelet aggregation caused by thrombin, weaken the coagulation process, reduce blood viscosity, improve collateral circulation, and restore and unblock blood [40]. The heart is a very important organ in blood circulation. Poor blood circulation will have a significant impact on the heart. Therefore, in this study, in addition to measuring the tail of mice, this experiment also measured the expression of these two proteins in heart tissue. This study also confirmed that thrombosis has a significant effect on the expression of these two important coagulation factors. The LDSB-KSFY07 can effectively inhibit this abnormality, so as to regulate the abnormal coagulation and promote the recovery of mice to normal state.

In the pathological state of thrombosis, the cell wall components of the intestinal flora enter the blood circulation and produce endotoxaemia, which can promote the production of a large number of free radicals in the body that cause organ and tissue damage. It is currently believed that this inflammation can play an important role in the pathogenesis of thrombosis [41]. Body damage caused by intestinal dysbiosis is often accompanied by high coagulability, which can increase the content of coagulation factors and reduce plasminogen activity [42]. The human gut flora is mainly composed of *Firmicutes* and *Bacteroides*, which account for more than 90% of the human gut flora. The presence of more *Firmicutes* microorganisms in the gut than *Bacteroides* microorganisms will increase the blood viscosity and the possibility of coagulation and greatly increase the probability of thrombosis compared with the normal state [43]. *Lactobacillus* and *Bifidobacterium* are both probiotics with good activity that can effectively help the body maintain the normal metabolism and discharge of toxic substances, and they also play a role in regulating blood circulation and thus can reduce the inflammation caused by various factors in the body and the possibility of thrombosis [44]. Healthy gut flora can reduce the risk of thrombosis by various mechanisms, such as regulating platelet function and changing coagulation function [45]. Kappa-carrageenan can break the intestinal lysosomal membrane and cause intestinal inflammation. Intestinal inflammation will have a significant impact on the intestinal flora. At the same time, after kappa-carrageenan causes thrombosis, the blood circulation in mice is not smooth, and the impact on immunity will also indirectly affect the intestinal flora [46]. In clinical studies, gut microbiome-derived metabolites have been shown to predict arterial thrombosis. Gut microbes can also synthesize substances that affect platelets through serotonin and may enhance von Willebrand factor production [47]. A study of elderly patients found that patients with probiotic deficiency in the gut exhibited higher inflammatory status, hypoalbuminemia, and severe portal vein thrombosis (PVT) [48]. These studies showed that gut microbes were closely related to thrombosis, and interventions in gut microbiota might play a key role in thrombosis. This study also confirmed that LDSB-KSFY07 can promote the production of more *Bacteroides* and *Bifidobacterium* microorganisms, supplemented *Lactobacillus* bacteria in the mouse gut, and reduced *Firmicutes*, thus inhibiting inflammation in the body, promoting normal blood circulation, and exerting an inhibitory effect on thrombosis. The side effects of heparin include nausea, vomiting, and allergy. LDSB-KSFY07, a lactic acid bacteria isolated from food, has no side effects and is a safe and effective biological agent; thus, it has important application prospects. In addition, the sequencing results of this study also show that LDSB-KSFY07 is a newly discovered lactic acid bacteria, and it comes from the original ecological pastoral areas that are not affected by industrialization, so it has good development and utilization value.

## 5. Conclusion

In this study, a newly discovered lactic acid bacterium (LDSB-KSFY07) was applied in a mouse thrombosis model, and its inhibitory effect on thrombosis was observed in animal experiments. The experimental results showed that LDSB-KSFY07 can effectively regulate the degree of black tail and blood clots in mice and the level of oxidation-related indicators in the serum and liver tissues, thus exerting oxidative stress inhibition and avoiding inflammation and damage caused by free radicals in organogenesis. Further assays demonstrated that LDSB-KSFY07 was able to regulate the mRNA expression of the NF-*κ*B pathway and proinflammatory factors, which reduced thrombosis caused by oxidative stress-induced inflammatory responses. Moreover, this study further showed that LDSB-KSFY07 is sufficient to regulate the mouse intestinal microorganism and improve the health of the intestinal microbial community, which can improve the health of the body, reduce the degree of damage of free radicals, and exert an inhibitory effect on thrombosis. The experimental results showed that LDSB-KSFY07 has a good experimental thrombotic inhibition effect. However, results from human trials are still lacking; therefore, enhanced clinical research is needed in the future.

## Figures and Tables

**Table 1:** Primer sequence in this experiment.

Gene	Forward sequence	Reverse sequence
*NF-κB p65*	5′-GAGGCACGAGGCTCCTTTTCT-3′	5′-GTAGCTGCATGGAGACTCGAACA-3′
*ICAM-1*	5′-TCCGCTACCATCACCGTGTAT-3′	5′-TAGCCAGCACCGTGAATGTG-3′
*VCAM-1*	5′-TTGGGAGCCTCAACGGTACT-3′	5′-GCAATCGTTTTGTATTCAGGGGA-3′
*E-selectin*	5′-ATAACGAGACGCCATCATGC-3′	5′-TGTCCACTGCCCTTGTGC-3′
*Cu/Zn-SOD*	5′-AACCAGTTGTGTTGTCAGGAC-3′	5′-CCACCATGTTTCTTAGAGTGAGG-3′
*Mu/Zn-SOD*	5′-CAGACCTGCCTTACGACTATGG-3′	5′-CTCGGTGGCGTTGAGATTGTT-3′
*GSH-Px*	5′-GTGCAATCAGTTCGGACACCA-3′	5′-CACCAGGTCGGACGTACTTG-3′
*PAI-1*	5′-TGCTTTTCTCTCTCTCCCTCTTTC-3′	5′-ACCAACAAAATTCAAGACCATGTG-3′
*t-PA*	5′-ACATGAAGCAATGACAAAGAAAGC-3′	5′-GCTCACGAAGCTGATGGTGTAAAG-3′
*Total bacteria*	5′-ACTCCTACGGGAGGCAGCAGT-3′	5′-ATTACCGCGGCTGCTGGC-3′
*Firmicutes*	5′-GCGTGAGTGAAGAAGT-3′	5′-CTACGCTCCCTTTACAC-3′
*Bacteroidetes*	5′-ACGCTAGCTACAGGCTTAACA-3′	5′-ACGCTACTTGGCTGGTTCA-3′
*Lactobacillus*	5′-CACCGCTACACATGGAG-3′	5′-AGCAGTAGGGAATCTTCCA-3′
*Bifidobacterium*	5′-TCGCGTCYGGTGTGAAAG-3′	5′-CCACATCCAGCRTCCAC-3′
*GAPDH*	5′-TGACCTCAACTACATGGTCTACA-3′	5′-CTTCCCATTCTCGGCCTTG-3′

**Table 2:** The activated partial thromboplastin time (APTT), thrombin time (TT), fibrinogen (FIB), and prothrombin time (PT) of mice with kappa-carrageenan-induced thrombosis.

Group	APTT (s)	TT (s)	FIB (g/L)	PT (s)
Normal	152.75 ± 10.86^a^	35.63 ± 4.21^d^	62.38 ± 3.25^d^	5.38 ± 1.51^d^
Model	75.38 ± 6.65^d^	82.00 ± 5.53^a^	107.25 ± 8.21^a^	21.75 ± 1.28^a^
Heparin	126.63 ± 8.88^b^	51.25 ± 3.54^b^	72.00 ± 4.66^b^	11.25 ± 1.04^c^
LDSB-KSFY07-L	93.63 ± 6.99^c^	64.63 ± 3.96^c^	90.25 ± 6.41^c^	16.25 ± 0.71^b^
LDSB-KSFY07-H	119.75 ± 7.78^b^	55.25 ± 3.85^b^	76.25 ± 5.92^b^	12.75 ± 0.89^c^

The results have been expressed as mean ± deviation. ^a–d^Mean values with different letters in the different column are significantly different (*P* < 0.05) according to Duncan's multiple range test.

**Table 3:** Serum SOD, CAT, and GSH-Px enzyme activities and MDA level of mice with kappa-carrageenan-induced thrombosis.

Group	T-SOD (U/mL)	CAT (U/mL)	MDA (mmol/mL)	GSH-Px (U/mL)
Normal	420.47 ± 21.36^a^	90.39 ± 6.33^a^	16.85 ± 4.12^d^	1302.58 ± 52.35^a^
Model	132.69 ± 18.32^d^	25.95 ± 4.96^d^	88.23 ± 6.32^a^	308.63 ± 20.16^d^
Heparin	333.64 ± 31.26^b^	71.35 ± 6.69^b^	39.78 ± 5.31^c^	922.36 ± 42.37^b^
LDSB-KSFY07-L	207.37 ± 25.60^c^	40.10 ± 5.87^d^	66.99 ± 5.12^b^	239.86 ± 32.06^c^
LDSB-KSFY07-H	297.89 ± 27.82^b^	68.26 ± 6.22^b^	42.99 ± 4.77^c^	897.89 ± 44.38^b^

The results have been expressed as mean ± deviation. ^a–d^Mean values with different letters in the different column are significantly different (*P* < 0.05) according to Duncan's multiple range test.

## Data Availability

The original contributions presented in the study are included in the article/supplementary material; further inquiries can be directed to the corresponding authors.
